# Multigene Phylogeny and Morphology Reveal Three Novel Species and a Novel Record of *Agaricus* From Northern Thailand

**DOI:** 10.3389/fmicb.2021.650513

**Published:** 2021-06-21

**Authors:** Chanyawat Jaichaliaw, Jaturong Kumla, Santhiti Vadthanarat, Nakarin Suwannarach, Saisamorn Lumyong

**Affiliations:** ^1^MS Degree Program in Applied Microbiology, Department of Biology, Faculty of Science, Chiang Mai University, Chiang Mai, Thailand; ^2^Department of Biology, Faculty of Science, Chiang Mai University, Chiang Mai, Thailand; ^3^Research Center of Microbial Diversity and Sustainable Utilization, Faculty of Science, Chiang Mai University, Chiang Mai, Thailand; ^4^Academy of Science, The Royal Society of Thailand, Bangkok, Thailand

**Keywords:** Agaricales, new taxa, phylogeny, saprophytic mushroom, taxonomy

## Abstract

*Agaricus* is a saprophytic mushroom genus widely distributed throughout the world. In this study, a survey of the *Agaricus* species carried out around Chiang Mai University in northern Thailand from 2018 to 2019 yielded 12 collections. Morphological characteristics and phylogenic analyses based on the internal transcribed spacers (ITS) and a fragment of the large subunit (LSU) of the nuclear ribosomal DNA (rDNA), and a fragment of the translation elongation factor 1-alpha (*tef*1) genes were investigated. The results revealed that these collections belong to six species including *Agaricus erectosquamosus*, *Agaricus pallidobrunneus*, *Agaricus subrufescens*, and three new species. *Agaricus thailandensis* sp. nov. was found to belong to *Agaricus* sect. *Minores*, which is placed in *Agaricus* subg. *Minores. Aagricus pseudoerectosquamosus* sp. nov. was placed in *Agaricus* sect. *Brunneopicti* within *Agaricus* subg. *Pseudochitonia.* Furthermore, *Agaricus lannaensis* remains an incertae sedis in *Agaricus* subg. *Pseudochitonia*. Additionally, this study was proposed that *A. pallidobrunneus* was discovered in Thailand for the first time. Full descriptions, color photographs, illustrations, and phylogenetic trees are provided.

## Introduction

The genus *Agaricus* L. with *Agaricus campestris* L. as the type species was first proposed by Donk (1962). This genus belongs to the family Agaricaceae of the order Agaricales. It is distributed worldwide and is commonly found in grasslands and forests (Kerrigan et al., 2005, Kerrigan, 2016; Parra, 2008, 2013; Zhao et al., 2012a, 2016; Chen et al., 2015; He et al.,2018a,b). *Agaricus* is a saprophytic fungal genus and is characterized by its white to pink free lamellae when young, which becomes brown at maturity, the presence of an annulus on the stipe, and brown to dark brown spore prints (Kerrigan et al., 2005; Zhao et al., 2016; He et al., 2017, 2018a). In the global market, *Agaricus bisporus* (J.E. Lange) Imbach (white bottom mushroom), and *Agaricus subrufescens* Peck (almond mushroom) are edible mushrooms that are commercially cultivated. Medicinal properties have been reported for both species (He et al., 2017, 2018a; Zhang et al., 2017). Many other *Agaricus* species such as *Agaricus agrinferus* (Kerrigan and Callac), *Agaricus augustus* Fr., *Agaricus bitorquis* (Quél.) Sacc., *A. campestris, Agaricus flocculosipes* (Zhao, Desjardin, Guinb, and Hyde), *Agaricus sinodeliciosus* (Zhao, Wang, and Zhao), and *Agaricus taeniatus* (Sai, Li, Shao, Li, and Wen) have been reported as edible (Kerrigan et al., 2008; Zhao et al., 2012b; Li et al., 2014; Thongklang et al., 2014b; Wang et al., 2015; Karunarathna et al., 2016; Zhang et al., 2017). In contrast, *Agaricus* species in *Agaricus* sect. *Xanthodermatei* and *Agaricus* sect. *Hondenses* have been described as poisonous as they contain toxic phenolic compounds that can cause gastrointestinal symptoms (Kerrigan et al., 2005; Petrova et al., 2007; Parra, 2013; Asef et al., 2016).

There are approximately 6,000 records of *Agaricus* in the Species Fungorum (accessed in April 2021). However, these records have been found to include synonyms, some misidentifications, and a number of species that have not yet been well-documented. To date, a total of over 500 species of *Agaricus* are currently recognized. These include many new species from America, Asia, Australia, and Europe (Wang et al., 2015; Kerrigan, 2016; He et al., 2017; Callac and Chen, 2018) and few new species from Africa (Hama et al., 2010; Zhao et al., 2012b; Ling et al., 2021) and Oceania (Geml et al., 2007; Lebel and Syme, 2012; Lebel, 2013). Among the approximately 200 new species described since 2000, more than half have been reported to be from Asia, and a quarter have been reported in America (particularly North America) (Zhao et al., 2011, 2016; Thongklang et al., 2014a; Liu et al., 2015; Karunarathna et al., 2016; Kerrigan, 2016; Chen et al., 2017; Callac and Chen, 2018; Hyde et al., 2018). Morphological characteristics, odor, and the chemical reactions of Schäffer’s reagent and KOH have mainly been used in the traditional identification of the *Agaricus* species (Parra, 2008; Karunarathna et al., 2014; Chen et al., 2015, 2017; Zhou et al., 2016). However, high variations of phenotypes, varying environmental factors, differing geographic conditions, and the separate developmental stages of basidiomata may influence the morphological identification process. This could make it difficult to distinguish this particular species from other closely related *Agaricus* species (Heinemann, 1978; Kerrigan, 1986; Singer, 1986; Callac et al.,1998a,b; Challen et al., 2003; Parra, 2008). Therefore, the application of molecular tools that are based on DNA analyses has proven to be essential in identifying the *Agaricus* species (Challen et al., 2003; Kerrigan et al., 2005, 2008; Zhao et al., 2011; Chen et al., 2015; Thongklang et al., 2016).

Zhao et al. (2016) classified *Agaricus* into five subgenera and 20 sections based on morphological characteristics, multigene molecular phylogeny (ITS, LSU, and *tef*1), and the divergence time. The revised classification of *Agaricus* by Chen et al. (2017), and Parra et al. (2018) resulted in six subgenera (*Agaricus* subg. *Agaricus*, *Agaricus* subg. *Flavoagaricus*, *Agaricus* subg. *Minores*, *Agaricus* subg. *Minoriosis*, *Agaricus* subg. *Pseudochitonia* and *Agaricus* subg. *Spissicaules*) with 23 sections. Subsequently, a new section of *Agaricus* subg. *Pseudochitonia* was introduced by He et al. (2018a). Therefore, *Agaricus* now contains six subgenera and 24 sections.

Over the last decade, the study of *Agaricus* has expanded rapidly, especially in tropical regions. Notably, Thailand is proving to be a hot spot for the discovery of novel species. This is evidenced by the discovery of many new species of macrofungi, 45 of which are new *Agaricus* species that have been discovered since 2011 (Zhao et al., 2011, 2012a, 2016; Karunarathna et al., 2014; Thongklang et al.,2014a,b; Ariyawansa et al., 2015; Chen et al., 2015, 2017; Liu et al., 2015; Li et al., 2016; Hyde et al., 2017, 2018; He et al., 2018a). This study outlines how we found twelve *Agaricus* specimens during the course of our investigation of macrofungi in northern Thailand. Among these, we describe three new species and one new record. This investigation introduces the taxa based on studies of morphology and multigene analyses of combined ITS, LSU, and *tef*1 sequences. Additionally, this study included a mini-review of *Agaricus* species that are found in Thailand.

## Materials and Methods

### Sample Collection

*Agaricus* were surveyed at Chiang Mai University, Chiang Mai Province, Thailand during the rainy seasons of the years 2018 and 2019. Photographs were immediately taken in the field. Basidiomata were collected and wrapped in aluminum foil and kept in plastic boxes while being transferred to the laboratory within 24 h of collection. Specimens were dried in a hot air oven at 45°C until they were completely dried. They were then kept in a plastic zip-locked bag and deposited in the Biology Department’s Herbarium (CMUB) along with the Herbarium of Sustainable Development of Biological Resources (SDBR-CMU), Faculty of Science, Chiang Mai University, Thailand. Facesoffungi and MycoBank numbers have also been provided (Robert et al., 2013; Jayasiri et al., 2015).

### Morphological Observation

Macroscopic descriptions were made based on fresh specimens. Color, name, and codes were given according to the methods employed by Kornerup and Wanscher (1978). Chemical reactions were determined following the methods described by Chen et al. (2015) and He et al. (2017) including Melzer’s reagent, 10% potassium hydroxide (KOH) in water, and Schäffer’s reaction. Microscopic characteristics, including basidiospores, basidia, cystidia, and pileipellis, were observed from dried specimens that had been rehydrated in 95% ethanol followed by distilled water, 5% aqueous KOH, or Melzer’s reagent. A minimum of 50 basidiospores, 20 basidia, and cystidia were measured using a compound light microscope (Olympus CX31, Japan). Basidiospores are presented in the following format: (*a*)*b*–*c*–*d*(*e*), for which “*c*” represents the average, “*b*” and “*d*” represent the average + and - standard deviation (SD), respectively, and “*a*” and “*e*” represent the minimum and maximum values, respectively. For spore statistics, Q represents the ratio of length divided by the width of the basidiospore, and Q_*m*_ represented the average Q of all specimens ± standard deviation.

### DNA Extraction, PCR Amplification, and Sequencing

DNA was extracted from the fresh tissue of each specimen using the FAVOGEN Genomic DNA Extraction Mini Kit (Taiwan) by following the manufacturer’s instructions. The ITS region of the rDNA was amplified using the ITS4/ITS5 primers (White et al., 1990) by polymerase chain reaction (PCR). The LSU of the rDNA gene was amplified with LR5/LROR primers (Vilgalys and Hester, 1990) and the *tef*1 gene was amplified with primers EF1-983F/EF1-1567R (Rehner and Buckley, 2005). The PCR programs of ITS, LSU, and *tef*1 genes were established by following the methods employed by He et al. (2017). PCR products were checked by electrophoresis on 1% agarose gels stained with ethidium bromide and observed under UV light. PCR products were purified using NucleoSpin Gel and a PCR Clean-up Kit (Macherey-Nagel, Germany). PCR products were then sent to a commercial sequencing provider (1^*ST*^ BASE Company, Kembangan, Malaysia). The obtained sequences were ultimately subjected to BLASTn search in GenBank.^1^

### Sequence Alignment and Phylogenetic Analyses

Newly generated sequences were assembled using the Sequencher program. Details of the sequences used for phylogenetic analysis obtained from this study and previous other studies are provided in Supplementary Table 1. Two sequence datasets were prepared for phylogenetic analyses. The first dataset was comprised of sequences of two subgenera, namely *Agaricus* subg. *Flavoagaricus* and *Agaricus* subg. *Minores*. The second dataset contains sequences of six sections in *Agaricus* subg. *Pseudochitonia* namely, *Agaricus* sect. *Bohusia*, *Agaricus* sect. *Brunneopicti*, *Agaricus* sect. *Floculenti*, *Agaricus* sect. *Nigrobrunnescentes*, *Agaricus* sect. *Rubricosi*, and *Agaricus* sect. *Sanguinolenti*. The datasets were then aligned using MAFFT version 7 (Katoh and Standley, 2013). The first and second aligned datasets were deposited in TreeBASE under the numbers 27426 and 28087, respectively. Maximum Likelihood (ML) phylogenetic tree inference was performed for each dataset using RAxML-HPC2 version 8.2.10 (Stamatakis, 2006) on the CIPRES web portal (Miller et al., 2009). The phylogenetic tree was inferred from a four-partitions (ITS, LSU, *tef*1 exons, and *tef*1 introns) combined dataset using the GTRCAT model with 25 categories. *A. campestris* LAPAG370 was used as an outgroup for both datasets. Statistical support of the clades was obtained with 1,000 rapid bootstrap replicates. For Bayesian Inference (BI), the best-fit model of substitution amongst those implementable in MrBayes was estimated separately for each region using jModelTest 2 (Darriba et al., 2012) on the CIPRES portal based on the Bayesian Information Criterion (BIC). The selected models that are similar in both data sets, were HKY + I + G for ITS, SYM + G for *tef*1 exons, K80 + G for introns of *tef*1. While for LSU, were K80 + I + G in the first dataset, and K80 + I in the second dataset. Partitioned BI was performed with MrBayes 3.2.6 software for Windows (Ronquist et al., 2012). Two runs of five chains were conducted for eleven million (first dataset) and three hundred thousand generations (second dataset), which were sampled every 200 generations. At the end of the runs, the average deviations of split frequencies were 0.007058 (first dataset) and 0.009527 (second dataset). The potential scale reduction factor values of all parameters were close to 1. The burn-in phase (25%) was estimated by checking the stationarity in the plot generated by the sump command.

## Results

### Phylogenetic Analyses Results

**FIGURE 1:**
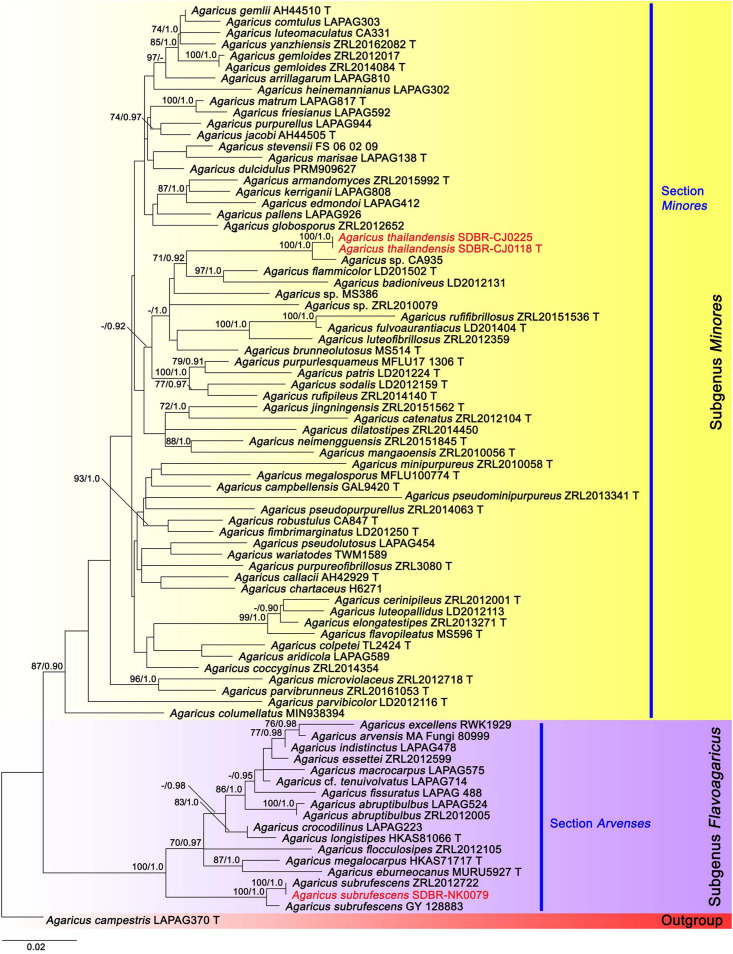
Phylogenetic tree of the genus *Agaricus* generated from maximum likelihood based on multigene sequences (ITS, LSU, *tef*1 exons, and *tef*1 introns) of *Agaricus* subg. *Flavoagaricus* and *Agaricus* subg. *Minores*. *Agaricus campestris* was used as an outgroup. The vouchers from this study are in red and “T” means type specimen. Bootstrap support values (BS ≥ 70%) and posterior probabilities (PP ≥ 0.90) are presented above the supported branches.

A total of 36 sequences from 12 specimens were newly obtained from this study and deposited in GenBank (Supplementary Table 1). The combined ITS, LSU, *tef*1 exons, and *tef*1 introns sequence datasets consisted of 81 and 54 taxa in the first and second datasets, respectively. The first and second aligned datasets were comprised of 2257 (ITS: 1–778, LSU: 779–1674, *tef*1 exons: 1675–2140 and *tef*1 introns: 2141–2257) and 2204 (ITS: 1–784, LSU: 785–1689, *tef*1 exons: 1690–2093 and *tef*1 introns: 2094–2204) characters including gaps, respectively. ML and BI trees of both datasets revealed similar topologies without any supported conflict (BS ≥ 70% and PP ≥ 0.90). Phylogenetic results were based on the first dataset (Figure 1) which contained *Agaricus* subg. *Flavoagaricus* and *Agaricus* subg. *Minores*. Selected *Agaricus* species formed two main clades namely *Agaricus* subg. *Flavoagaricus* (BS = 100%, PP = 1) and *Agaricus* subg. *Minores* (BS = 87%, PP = 0.90). *Agaricus thailandensis* (vouchers SDBR-CMU-CJ118 and SDBR-CMU-CJ225) formed a monophyletic clade (BS = 100%, PP = 1) in *Agaricus* sect. *Minores* of *Agaricus* subg. *Minores* and closely related to *Agaricus* sp. voucher CA935 with the high support values (BS = 100%, PP = 1). Furthermore, they formed a sister clade to *Agaricus flammicolor* (Chen et al., 2017) and *A*. *badioniveus* (Chen et al., 2017) (BS = 71%, PP = 0.92). Another obtained specimen voucher SDBR-NK0079 was placed within the cluster of the known species clade of *A*. *subrufescens* (BS = 100%, PP = 1) in *Agaricus* sect. *Arvenses* of *Agaricus* subg. *Flavoagaricus*.

**FIGURE 2:**
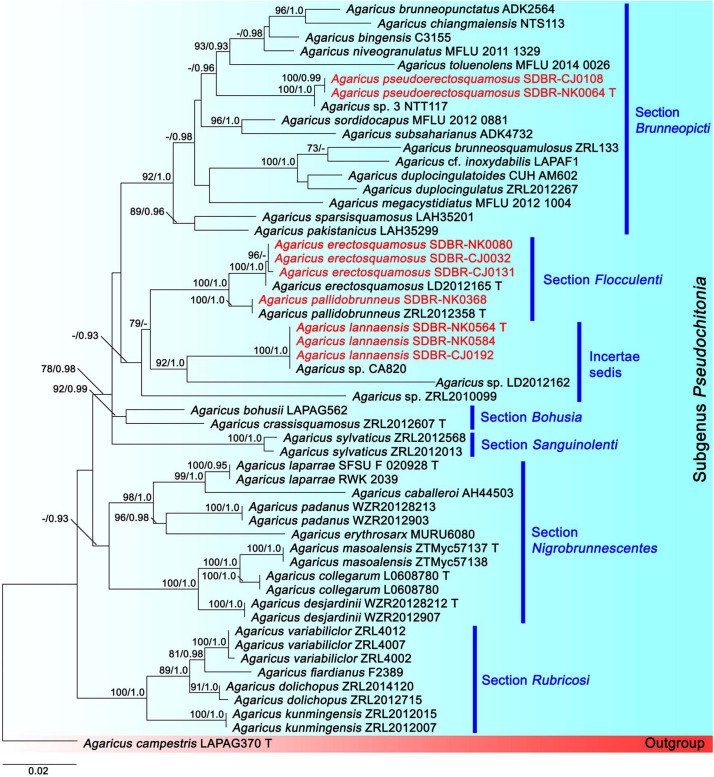
Phylogenetic tree of the genus *Agaricus* generated from maximum likelihood based on multigene sequences (ITS, LSU, *tef*1 exons, and *tef*1 introns) of six sections in *Agaricus* subg. *Pseudochitonia* namely, *Agaricus* sect. *Bohusia*, *Agaricus* sect. *Brunneopicti*, *Agaricus* sect. *Floculenti*, *Agaricus* sect. *Nigrobrunnescentes*, *Agaricus* sect. *Rubricosi* and *Agaricus* sect. *Sanguinolenti*. *Agaricus campestris* was used as an outgroup. The vouchers from this study are in red and “T” means type specimen. Bootstrap support values (BS ≥ 70%) and posterior probabilities (PP ≥ 0.90) are presented above the supported branches.

The second tree was based on sequences of six sections of *Agaricus* subg. *Pseudochitonia* (Figure 2). Six main clades were assigned including, *Agaricus* sect. *Bohusia* (BS = 92%, PP = 0.99), *Agaricus* sect. *Brunneopicti* (BS = 92%, PP = 1), *Agaricus* sect. *Floculenti* (BS = 62%, PP = 0.93), *Agaricus* sect. *Nigrobrunnescentes* (BS = 57%, PP = 0.93), *Agaricus* sect. *Rubricosi* (BS = 100%, PP = 1) and *Agaricus* sect. *Sanguinolenti* (BS = 100%, PP = 1). Two new species, namely *Agaricus lannaensis* and *Aagricus pseudoerectosquamosus* formed two different species clades within *Agaricus* subg. *Pseudochitonia*. *Agaricus lannaensis* (vouchers SDBR-CJ192, SDBR-NK0584, and SDBR-NK0564) formed a highly supported clade with *Agaricus* sp. voucher CA820 (BS = 100%, PP = 1) and they formed a sister clade to unnamed species *Agaricus* voucher LD2012162 (BS = 92%, PP = 1). This clade was closely related to the clade of *Agaricus* sect. *Flocculenti.* Another new species, *A*. *pseudoerectosquamosus* (vouchers SDBR-CJ108 and SDBR-NK0064) formed a clade (BS = 100%, PP = 0.99) that was closely related to *Agaricus* sp. voucher NTT117 with the high support values (BS = 100%, PP = 1), in *A*garicus sect. *Brunneopicti* (BS = 92%, PP = 1). Additionally, the other four *Agaricus* collections obtained in this study were clustered within two known species clades, in which *Agaricus* vouchers SDBR-NK0080, SDBR-CJ0032, and SDBR-CJ0131 were clustered with *Aagricus erectosquamosus* Linda J. Chen, K.D. Hyde & R.L. Zhao. *Agaricus* voucher SCBR-NK0368 formed a clade with *Agaricus pallidobrunneus* R.L. Zhao. with high value supports (BS = 100%, PP = 1).

### Taxonomic Description of New Species

**FIGURE 3:**
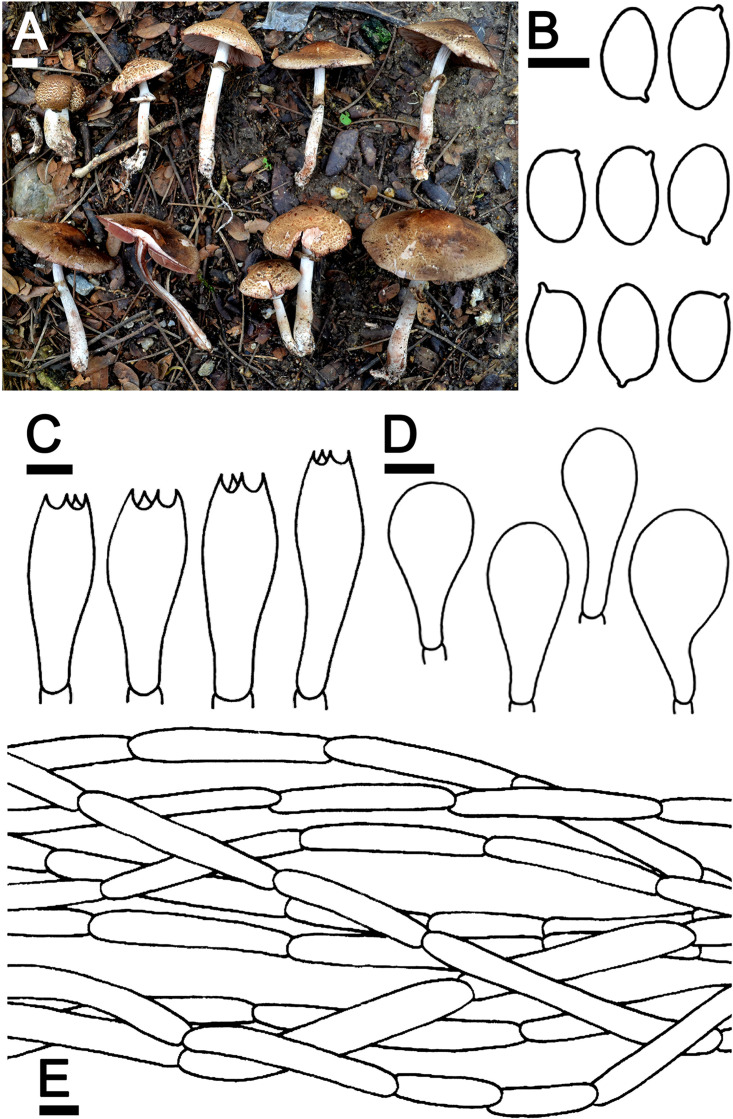
*Agaricus lannaensis* SDBR-NK0564 (holotype). **(A)** Basidiomata on the field, **(B)** Basidospores, **(C)** Basidia, **(D)** Cheilocystidia, **(E)** Pileipellis. Scale bars **(A)** = 1 cm and **(B–E)** = 5 μm.

***Agaricus lannaensis*** N. Suwannarach, J. Kumla & S. Lumyong sp. nov. Figure 3

MycoBank: MB 838052

Facesoffungi number: FoF 09468

Etymology: “*lannaensis*” referring to the Kingdom of Lanna, the historic name of northern Thailand, where the new species was found.

Holotype: THAILAND, Chiang Mai Province, Mueang Chiang Mai District, Chiang Mai University, 18°47′33.6″N 98°57′30.2″E, elevation 331 m, June 2, 2019 (N. Suwannarach and J. Kumla) SDBR-NK0564, CMUB 39945.

Description: *Basidiomata* medium-sized, stipitate-pileate with lamellate hymenophore. *Pileus* (2.4) 3.2–7.4 cm in diameter, at first spherical, campanulate, then subumbonate with straight margins, and whole cap covered by fibrils and broken into small squamules. The surface background is pale orange (6A3) at the center and turns gradually orange white (5A2) at the margins, covered by innate scales. The pileus is at first covered, then gradually sprayed, denser at pileus, partially at margins when aged, appressed innately scaled, brown (7E8) at the top of the pileus, and gradually brown (7E7) at margins, then grayish red (8C5) when cut or touched. *Pileus context* soft white (1A1) then reddish brown (8E7) when cut, pluteoid. *Lamellae* emarginate, crowded, thin, regular, 3–4 different lengths of lamellae, at first pastel red (8A4), becoming dark brown (8F8). *Stipe* (4.3)4.6–8.8 × 0.6–1.2 cm, cylindrical, smooth above annulus until under lamellae, covered by small squamules which fibrilloses white (1A1) under annulus until the base, then grayish red (8C5) when cut or touched, scales reddish brown (8D5) at the base. *Stipe context* soft, fistulose, orange-white (6A2), then reddish brown (8E7) when cut. *Annulus* are membranous, thin, pendant, simple, stick above the middle of the stipe, at first white (1A1), dark brown (7F4) at edges, then light brown (7D5) when aged. *Spores print* dark brown (8F5) and the *odor* is phenol-like. Macrochemical reactions; KOH reaction yellow and Schäffer’s reactions negative.

*Basidiospores* (5.5)6.3–6.8–7.3(8.0) × (4.0)4.1–4.3–4.4(4.5) μm (*n* = 50), Q = 1.22–2.00, Q_*m*_ = 1.62 ± 0.19, broadly ellipsoid to elongate, smooth, thin-walled, brown in water and KOH, inamyloid. *Basidia* 19–26 × 5.5–8.5 μm, clavate, 4-spored, hyaline, sterigmata up to 2.5 μm long. *Cheilocystidia* 13–47 × 6.5–24 μm, clavate to broadly clavate, often with a short peduncle, hyaline. *Pleurocystidia* absent. *Pileipellis* composed of 3.8–7.5 μm wide cutis hyaline hyphae, smooth, cylindrical, occasionally branched. *Annulus* composed of 1–3 μm wide hyaline hyphae, cylindrical with rounded apex, branched. *Stipitipellis* composed of cutis hyphae wide up to 2–5 μm, cylindrical, occasionally branched, hyaline. *Clamp connections* absent.

Ecology and distribution: Fruiting solitary or gregarious on sandy loam soil during the rainy season (mid-May to October). Known only from Thailand.

Additional specimens examined: THAILAND, Chiang Mai Province, Mueang Chiang Mai District, Chiang Mai University, 18°47′33.6″N 98°57′30.2″E, elevation 331 m, June 2, 2019, J. Kumla, SDBR-NK0584; 18°47′45″N 98°57′2″E, elevation 340 m, October 9, 2019, C. Jaichaliaw, SDBR-CJ0192.

Note: In the field, *A. lannaensis* is morphologically similar to *Agaricus brunneopileatus* Callac & R.L. Zhao. However, *A. brunneopileatus* differs from *A. lannaensis* by the fact that it had a negative reaction in KOH and Schäffer’s reactions, smaller basidiospores (4.9–5.8 × 2.5–3.6 μm) and shorter basidia (12.6–18 × 6.2–8.8 μm) (Zhao et al., 2016). The phylogeny also supports the determination that they are different species, for which *A. brunneopileatus* was assigned to *Agaricus* sect. *Subrutilescentes* in *Agaricus* subg. *Spissicaules* (Zhao et al., 2016). Phylogenetically, *A. lannaensis* formed a clade with the unnamed species *Agaricus* sp. voucher CA820 collected from Thailand, and they formed a sister clade to *Agarius* sect. *Flocculenti* comprised with *A*. *erectosquamosus* and *A. pallidobrunneus* (Figure 2). *Agaricus erectosquamosus* were clearly distinguished from *A. lannaensis* by the orange KOH reaction and the presence of erect squamules on the pileus that appeared dense at the disc and brown against a dirty white background (Zhao et al., 2016). *A. pallidobrunneus* has brownish orange to dark brown pileus, but *A*. *lannaensis* has a pale orange pileus (Zhao et al., 2016).

**FIGURE 4:**
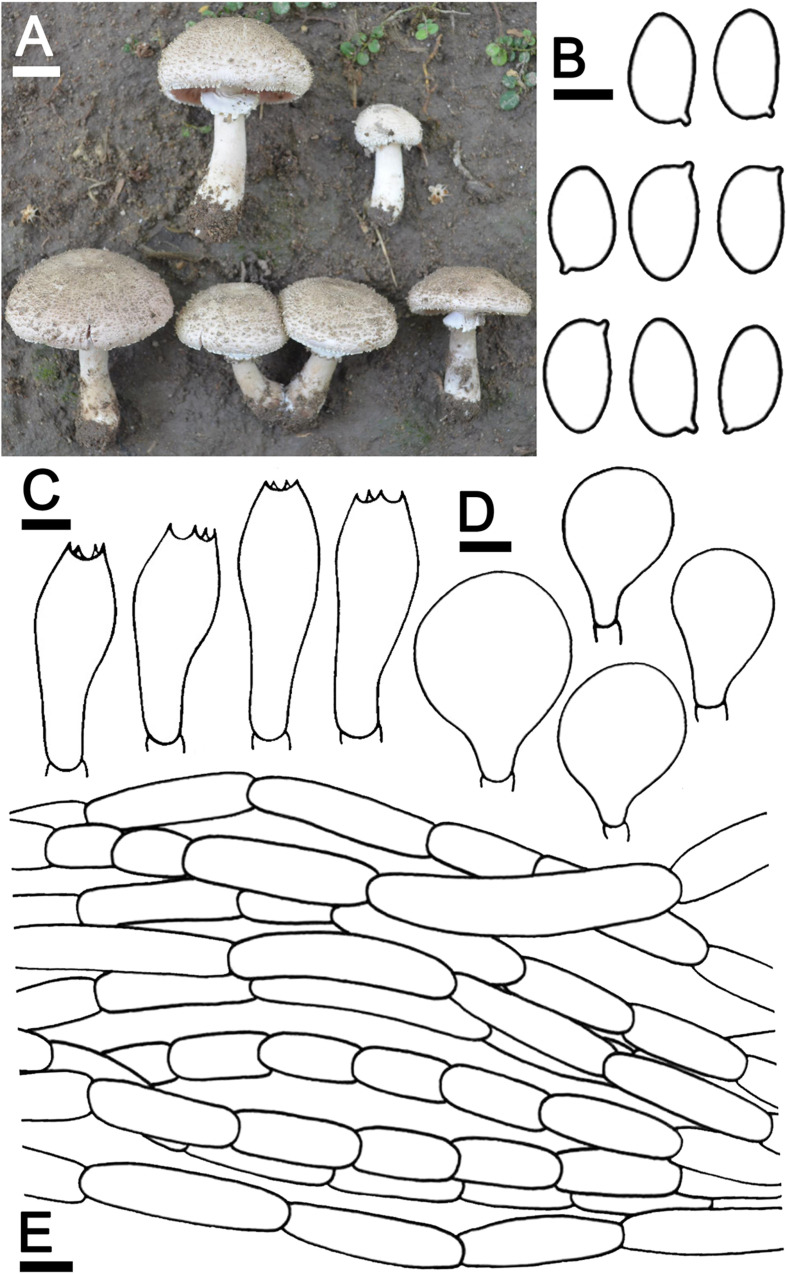
*Agaricus pseudoerectosquamosus* SDBR-NK0064 (holotype). **(A)** Basidiomata on the field, **(B)** Basidiospores, **(C)** Basidia, **(D)** Cheilocystidia, **(E)** Pileipellis. Scale bars **(A)** = 2 cm and **(B–E)** = 5 μm.

***Agaricus pseudoerectosquamosus*** J. Kumla, N. Suwannarach, and S. Lumyong sp. nov. (Figure 4)

MycoBank: MB 838053

Facesoffungi number: FoF 09469

Etymology: “*pseudo*” = false, referring to the morphological characteristics that are easily mistaken for *A. erectosquamosus*

Holotype: THAILAND, Chiang Mai Province, Mueang Chiang Mai District, Chiang Mai University, 18°48′6.4″N 98°57′23″E, elevation 330 m, September 20, 2018, J. Kumla, SDBR-NK0064, CMUB 39944

Description: *Basidiomata* medium-sized, stipitate-pileate with lamellate hymenophore. *Pileus* (3)5.2–7.5 cm in diameter, at first umbonate, broadly conical, then hemispherical with inflexed margin, rimos; surface background white (1A1), innately squamulose, denser at the center, poor at the margins with depressed at the center, uplifted, brownish orange (5C4) at the center, pale to white (1A1) at the edges. *Pileus context* soft, white (1A1). *Lamellae* free, crowded, even, 4–5 different lengths of lamellullae, pale red (8A3). *Stipe* 6.7–10 × 1.2–2.1 cm, subclavate, inserted or caespitose, surface skin covered pale yellow near lamellae to dark brown (4A3 to 6F8) at the base by the fibrillose, denser, white (1A1). *Annulus* thick, double, stick above the middle of stipe, white (1A1), and often cogwheel-like. *Stipe context* fistulose, white (1A1). *Spores print* dark brown (8F5). *Odor* phenol-like. Macrochemical reactions; KOH reaction yellow and Schäffer’s reactions negative.

*Basidiospores* (8.0)8.2–8.5–8.8(9.0) × (4.5)4.7–5.0–5.4(5.5) μm (*n* = 50), Q = 1.45–2.00, Q_*m*_ = 1.69 ± 0.14, ellipsoid to elongate, smooth, thin-walled, brown in water and KOH, inamyloid. *Basidia* 19.5–26 × 8–9.5 μm, clavate, 4-spored, hyaline, sterigmata up to 2 μm long. *Cheilocystidia* 19.5–40 × 11.5–22.5 μm, clavate to broadly clavate, often with a long peduncle, hyaline. *Pileipellis* composed of fibrilloses 5–20 × 4–5.6 μm hyaline hyphae, smooth, short cylindrical, occasionally branched, and cutis hyphae 2–5 μm, wide, occasionally branched, hyaline. *Annulus* composed of 2–4.5 μm wide hyaline hyphae, cylindrical with a rounded apex and branched. *Stipitipellis* composed of cutis hyphae up to 3–7 μm wide, cylindrical, occasionally branched, hyaline. *Clamp connections* absent.

Ecology and distribution: Fruiting solitary or gregarious on sandy loam soil during the rainy season (mid-May to October). Known only from Thailand.

Additional specimens examined: THAILAND, Chiang Mai Province, Mueang Chiang Mai District, Chiang Mai University, 18°48′17″N 98°57′13″E, elevation 340 m, September 22, 2019, C. Jaichaliaw, SDBR-CJ0108.

Note: Morphological characteristics and phylogenetic analysis assigned *A*. *pseudoerectosquamosus* to *Agaricus* sect. *Brunneopicti* in *Agaricus* subg. *Pseudochitonia*. Morphologically, *A*. *pseudoerectosquamosus* is similar to *A. erectosquamosus* (Zhao et al., 2016). However, the orange associated with the KOH reaction, the dark brown squamules on the surface of the pileus, the shorter basidiospores (6.6–7.6 × 4.1–5.0 μm) and the narrower basidia (15–22 × 6.5–7.5 μm) in *A. erectosquamosus* clearly distinguished it from *A. pseudoerectosquamosus*. Moreover, the phylogeny placed *A. erectosquamosus* within *Agaricus* sect. *Flocculenti* of *Agaicus* subg. *Pseudochitonia*, (Zhao et al., 2016). Phylogenetically, *A*. *pseudoerectosquamosus* is a monophyletic clade that formed a sister clade to the unnamed species *Agaricus* sp. 3 voucher NTT117 collected from Thailand, and they formed a sister clade to *A. bingensis* (Heinem., Bull. Jard. Bot. État Bruxelles), *A. brunneopunctatus* Linda (J. Chen, Callac, & Parra), *A. chiangmaiensis* (Karunarathna, Guinb. & Hyde), *A. niveogranulatus* (Linda J. Chen, R.L. Zhao, Callac & Hyde), and *A. toluenolens* (Callac, Linda J. Chen & K.D. Hyde) (Figure 2). Notably, these have all been found in Thailand except for *A. bingensis* and *A. brunneopunctatus*, which has been identified in Africa (Benin, Congo, Togo, and Uganda) (Pegler, 1977; Chen et al., 2015). However, *A. bingensis* has a larger pileus (8–25 cm in diameter), a longer stipe (11–20 × 1.2–4 cm), and the narrower basidia (16–21 × 5–6 μm) than *A*. *pseudoerectosquamosus* (Pegler, 1977; Chen et al., 2015). While, *A. brunneopunctatus* has mostly shorter basidiospores (7.3–8.45 μm) than *A*. *pseudoerectosquamosus* (Chen et al., 2015). *Agaricus chiangmaiensis* differs from *A*. *pseudoerectosquamosus* by its larger pileus (10–17 cm in diameter), narrower basidiospores (7–8.5 × 3–4 μm), and smaller cheilocystidia (15–18 × 8–10 μm) (Karunarathna et al., 2014). The pileus (10–16 cm in diameter) and basidia (16–24 × 6.8–8.5 μm) of *A. niveogranulatus* were larger and mostly narrower than *A*. *pseudoerectosquamosus*, respectively (Chen et al., 2015). Additionally, *A. toluenolens* mostly has a narrower stipe (3.5 × 0.6–1 cm) and basidia (16–21 × 7–8.5 μm) than *A*. *pseudoerectosquamosus* (Chen et al., 2015).

**FIGURE 5:**
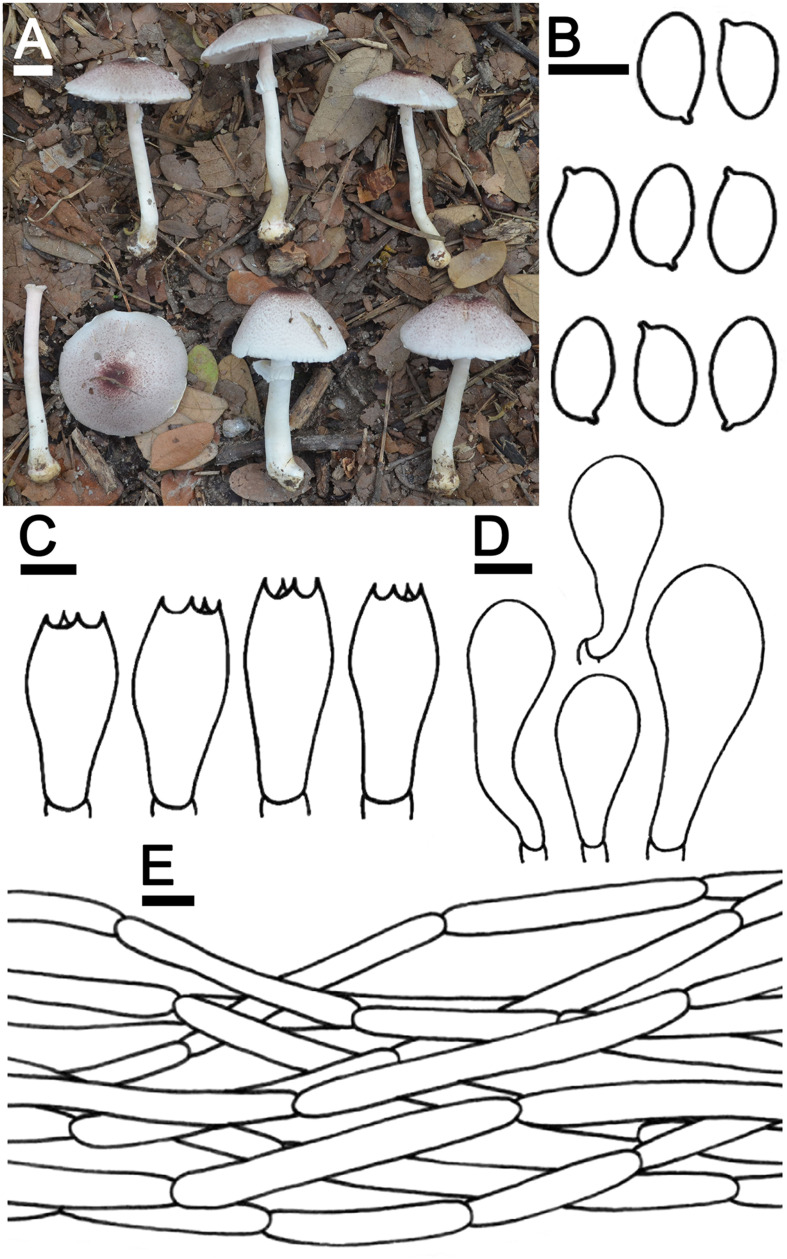
*Agaricus thailandensis* SDBR-CJ0118A (holotype). **(A)** Basidiomata on the field, **(B)** Basidiospores, **(C)** Basidia, **(D)** Cheilocystidia, **(E)** Pileipellis. Scale bars **(A)** = 1 cm, **(B–D)** = 5 μm, and **(E)** = 10 μm.

***Agaricus thailandensis*** Jaichliaw & S. Lumyong sp. nov. Figure 5

MycoBank: MB 838054

Facesoffungi number: FoF 09470

Etymology: “*thailandensis*” referring to Thailand, where the new species was found.

Holotype: THAILAND, Chiang Mai Province, Mueang Chiang Mai District, Chiang Mai University, 18°48′31″N 98°57′15″E, elevation 340 m, September 16, 2019, C. Jaichaliaw. SDBR-CJ0118, CMUB 39946

Description: *Basidiomata* medium-sized, stipitate-pileate with lamellate hymenophore. *Pileus* 2.6–5.8 cm in diameter, at first obtusely conical, then convex or plano-convex with straight margins; surface background white (1A1) to pinkish-white (13A2) at the center, covered by dark ruby (12F8) at the center, pale to grayish magenta (13D3) at the margins of the fibrillose scales, appressed, denser at the center of pileus and poor at the margin. *Pileus context* soft, white (1A1). *Lamellae* free, crowded, even, regular, thin, 2–3 different lengths of lamellae, concolorous, at first white (1A1), then reddish gray (12B2). *Stipe* 5.2–5.9 × 0.3–0.7 cm, cylindrical with subbulbous, inserted; surface smooth, background white (1A1) to dark yellow (4C8) at the base, covered by small fibrilloses, minutely, white (1A1). *Annulus* thin, simple, stick out above the middle of the stipe and at the margins of the pileus, pendant, white (1A1). *Stipe context* soft, fistulose, white (1A1). *Spores print* dark brown (7F8). The *odor* smells like almonds. Macrochemical reactions; KOH reaction yellow and Schäffer’s reactions orange.

*Basidiospores* (6.0)6.1–6.3–6.4(6.5) × (3.5)3.7–4.0–4.3(4.5) μm (*n* = 50), Q = 1.44–1.86, Q_*m*_ = 1.58 ± 0.15, ellipsoid to elongate, smooth, thin-walled, brown in water and KOH, inamyloid. *Basidia* 13–20 × 6–8 μm, clavate, 4-spored, hyaline, sterigmata up to 4 μm long. *Cheilocystidia* 12.5–50 × 7–15.5 μm, clavate to broadly clavate, often with a long peduncle, hyaline. *Pleurocystidia* absent. *Pileipellis* cylindrical hyphae 1.5–8.5 μm wide, elongate, constricted, smooth, occasionally branched, hyaline. *Annulus* composed of 2.5–21.5 μm wide, cylindrical with rounded apex, branched hyaline hyphae. *Stipitipellis* composed of 2.5–22.5 μm wide, cylindrical with rounded apex, constricted, hyaline. *Clamp connections* absent.

Ecology and distribution: Fruiting solitary or gregarious on sandy loam soil during the rainy season (mid-May to October). Known only from Thailand.

Additional specimens examined: THAILAND, Chiang Mai Province, Mueang Chiang Mai District, Chiang Mai University, 18°47′45″N 98°57′2″E, elevation 340 m, September 27, 2019 (C. Jaichaliaw) SDBR-CJ0225.

Note: *Agaricus thailandensis* was placed within *Agaricus* sect. *Minores* of *Agaricus* subg. *Minores* based on the morphological and molecular data. The grayish magenta to dark ruby fibrils on the pileus surface and the reaction of KOH Schäffer’s reactions of *A. thailandensis* are morphologically similar to those of *A. purpureofibrillosus* (Linda J. Chen, R.L. Zhao & K.D. Hyde) in *Agaricus* sect. *Minores* of *Agaricus* subg. *Minores* (Fr.) (Chen et al., 2017). In contrast, *A. purpureofibrillosus* has shorter basidiospores (4.5–5.3 × 2.7–3 μm) than *A. thailandensis* and their clear separation was supported by the phylogeny (Chen et al., 2017). Phylogenetically, *A. thailandensis* formed a sister clade to the unnamed species *Agaricus* sp. voucher CA935 collected from Thailand, and they formed a sister clade to *A. flammicolor* and *A. badioniveus* (Figure 1). Both species have been collected from China and Thailand (Chen et al., 2017). Morphologically, *A. flammicolor* differed from *A. thailandensis* by the presence of orange bright fibrils on the pileus, smaller basidiospores (4.4–6.2 × 2.5–3.2 μm) and narrower basidia (12–16 × 5–6 μm) (Chen et al., 2017). *Agaricus badioniveus* is different from *A. thailandensis* by the yellowish-brown fibrils on the pileus and the mostly smaller basidiospores (5–6.2 × 3.1–3.8 μm) (Chen et al., 2017).

### Taxonomic Description of New Record

***Agaricus pallidobrunneus*** R.L. Zhao., Fungal Divers.: 33, 2016 Figure 6

**FIGURE 6:**
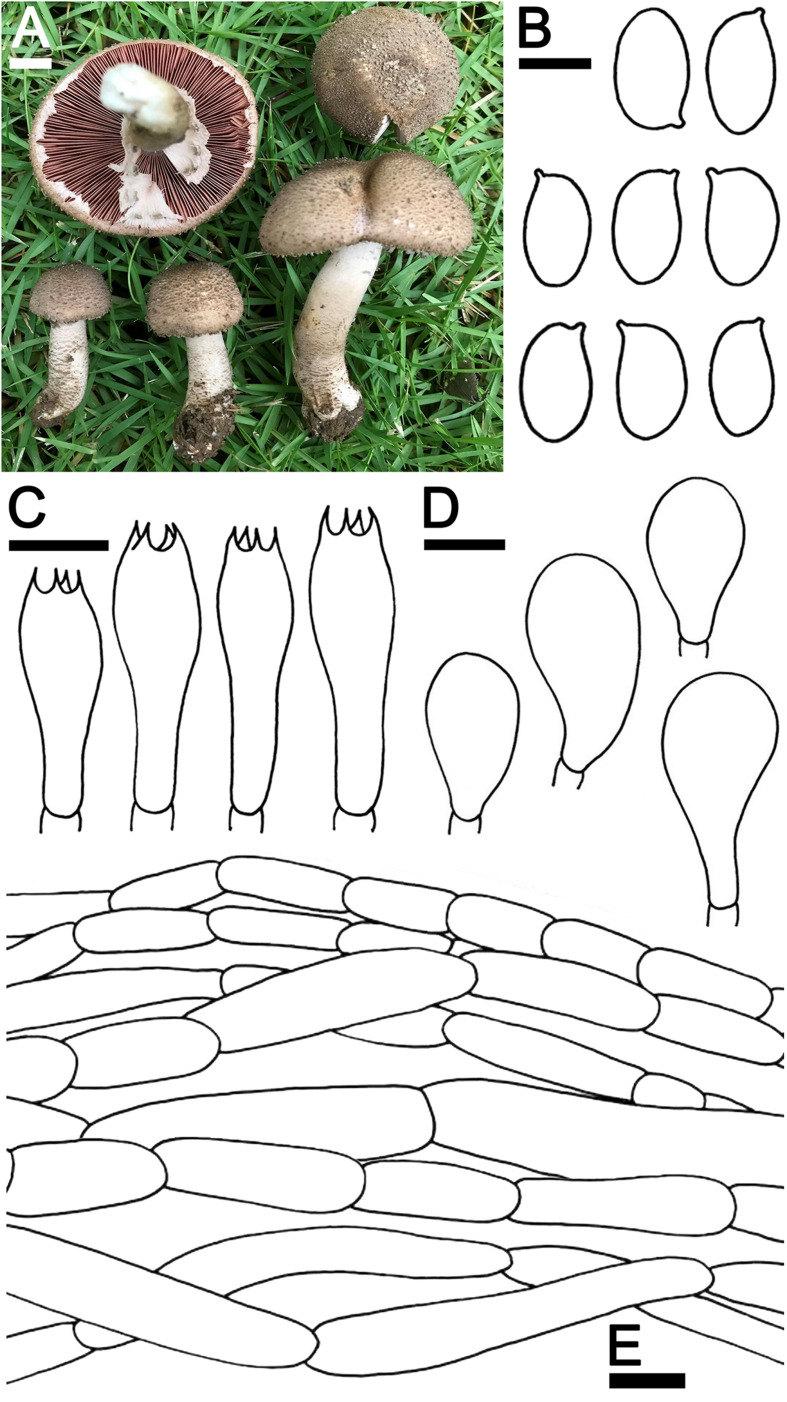
*Agaricus pallidobrunneus* SDBR-NK0368. **(A)** Basidiomata on the field, **(B)** Basidiospores, **(C)** Basidia, **(D)** Cheilocystidia, **(E)** Pileipellis. Scale bars **(A)** = 1 cm, **(B)** = 5 μm, and **(C–E)** = 10 μm.

Description: *Basidiomata* medium-sized, stipitate-pileate with lamellate hymenophore. *Pileus* (2)3.6–8 cm in diameter, paraboloid with inflexed margins, becoming applanate to plano-concave, depressed at the center with reflexed margins; surface background white (1A1), partially or entirely covered by brownish orange (5C5) to dark brown to (7F7) scales, the scales at first appressed then uplifted and innately scaled with age. *Pileus context* soft, white (1A1) grayish red (10D5) when cut. *Lamellae* free, crowded, thin, regular, 4–5 different lengths of lamellae, 3–4 mm wide half-way to margins, at first grayish rose (12B6) then grayish brown (7F3) when aged. *Stipe* 9–10 × 0.7–1.3 cm, tapering upwards, covered with small brown scales at the base, moist, white (1A1) near the cap, gradually light brown (5D4) at the base. *Annulus* thin, double, sticking up above the middle of the stipe and at margins, pendant, white (8A1) becoming brownish orange (5C5) when aged. *Stipe context* soft, fistulose, white (1A1), grayish red (10D5) when cut. *Spores print* dark brown (7F5). *Odor* is pleasant. Macrochemical reactions; KOH reaction yellow and Schäffer’s reactions negative.

*Basidiospores* (7)7.3–7.6–7.9(8.5) × (4.0)4.3–4.5–4.7(5.0) μm (*n* = 50), Q = 1.65–1.70, Q_*m*_ = 1.67 ± 0.08, ellipsoid to elongate, smooth, thin-walled, brown in water and KOH, inamyloid. *Basidia* 15–30 × 5–9 μm, clavate, 4-spored, hyaline, sterigmata up to 2.5 μm long. *Cheilocystidia* 18–29 × 10–17 μm, clavate to broadly clavate, hyaline. *Pleurocystidia* absent. *Pileipellis* composed of hyaline hyphae, smooth, short cylindrical, with occasionally branched and cutis hyphae 5–12.5 μm wide, occasionally branched, hyaline. *Annulus* composed of 5–9.5 μm wide hyaline hyphae, cylindrical with rounded apex, branched. *Stipitipellis* composed of cutis hyphae wide up to 2.5–7 μm, cylindrical, occasionally branched, hyaline. *Clamp connections* absent.

Ecology and distribution: Fruiting solitary or gregarious on sandy loam soil during the rainy season (mid-May to October). Known from China and Thailand.

Material examined: THAILAND, Chiang Mai Province, Mueang Chiang Mai District, Chiang Mai University, 18°48′5″N 98°57′24″E, elevation 330 m, October 31, 2018, J. Kumla, SDBR-NK0368.

Note: Morphological characteristics and phylogenetic analysis of Thai specimens were used to assign *A*. *pallidobrunneus* to *Agaricus* sect. *Brunneopicti* in *Agaricus* subg. *Pseudochitonia*, according to Zhao et al. (2016). *Agaricus pallidobrunneus* is morphologically similar to *A. erectosquamosus*. However, *A. erectosquamosus* differs from *A. pallidobrunneus* by its erect, dark brown squamules on the pileus and stipe surface. The phylogeny also supports the determination that they are different species (Zhao et al., 2016).

## Discussion

*Agaricus* is widely distributed in both temperate and tropical areas throughout the world (Kerrigan et al., 2005, Kerrigan, 2016; Chen et al., 2015; Zhao et al., 2016; He et al., 2017, 2018a,b; Callac and Chen, 2018; Ling et al., 2021). Morphological characteristics have been traditionally used in the identification of specimens of the *Agaricus* species. However, identification can be difficult as some species have similar features. Thus, identification can be limited by the morphological characteristics as well as the different environmental conditions that affect those morphological characteristics (Heinemann, 1978; Kerrigan, 1986; Singer, 1986; Callac et al.,1998a,b; Parra, 2008). Over the last two decades, molecular phylogeny has been an essential tool in the identification of *Agaricus* (Challen et al., 2003; Kerrigan et al., 2005, 2008; Zhao et al., 2011; Parra, 2013; Chen et al., 2015). The current classification of the genus *Agaricus* consists of six subgenera and twenty-four sections based on the combined data of morphological characteristics, the multigene phylogenetic analysis, and an estimation of divergence times (Zhao et al., 2016; Chen et al., 2017; He et al., 2018a; Parra et al., 2018).

In Thailand, the fourteen *Agaricus* species that were recorded by Thai taxonomists were identified only by their morphological characteristics. Of these, most have previously been found in temperate areas. However, there has been a lack of available molecular data on this species (Chandrasrikul et al., 2011) and there may have been incidences of mis-classification because tropical microflora are poorly understood. Thus, we are not sure these fourteen species have been accurately identified, expect *A. subrufescens* (Wisitrassameewong et al., 2012). *Agaricus* species richness is high in Thailand but many species have not yet been described based on a single collection and some of them have not been confirmed by phylogenetic data. Zhao et al. (2011) classified *Agaricus* collected from temperate and tropical regions, while ten *Agaricus* species collected from Thailand have been described based on their morphological and molecular characteristics.

During the period of 2012 to 2014, seven new species and three new records of *Agaricus* were found in Thailand (Chen et al., 2012; Wisitrassameewong et al., 2012; Zhao et al.,2012a,b; Karunarathna et al., 2014; Thongklang et al.,2014a,b). Thirty-seven new species of *Agaricus* were described from 2015 to 2017 (Ariyawansa et al., 2015; Chen et al., 2015, 2017; Liu et al., 2015; Thongklang et al., 2016; Zhao et al., 2016; Zhou et al., 2016; Hyde et al., 2017). In 2018, a new species of Thai *Agaricus* was reported by He et al. (2018a). Prior to this study, *Agaricus* in Thailand were classified in five subgenera (*Agaricus* subg. *Agaricus*, *Agaricus* subg. *Flavoagaricus*, *Agaricus* subg. *Minores*, *Agaricus* subg. *Pseudochitonia* and *Agaricus* subg. *Spissicaules*), thirteen sections and fifty-eight species based on morphological and molecular data.

In this study, six species of *Agaricus* including three new species (*A. lannaensis*, *A. pseudoerectosquamosus*, and *A. thailandensis*), one new record (*A. pallidobrunneus*), and two previously known species (*A. erectosquamosus* and *A. subrufescens*) collected from Chiang Mai Province, Thailand were identified based on their morphological characteristics and a multigene phylogenetic analysis. *Agaricus lannaensis* and *A. pseudoerectosquamosus* belong to *Agaricus* subg. *Pseudochitonia* in an incertae sedis clade and *Agaricus* sect. *Brunneopicti*, respectively. *Agaricus thailandensis* has been placed in *Agaricus* sect. *Minores* of *Agaricus* subg. *Minores*. Based on the phylogenetic analyses, *A. lannaensis* (including *Agaricus* sp. CA820) formed a sister clade to unnamed species *Agaricus* voucher LD2012162 (Figure 2); however, they cannot be assigned to *Agaricus* sect. *Flocculenti* according to the classification system of *Agaricus* (Zhao et al., 2016). *Agaricus* sp. CA820 collected from Thailand should be recognized in *A. lannaensis*, however, its morphological characteristics should be further confirmed. *Agaricus pseudoerectosquamosus* and *A. thailandensis* are closely related to the unnamed species *Agaricus* sp. voucher NTT117 and CA935, respectively which have been collected in Thailand (Figures 1, 2). However, there is a lack of available information on the morphological characteristics of these unnamed species. Thus, their species definition will be required in future studies. Notably, *A. subrufescens* and *A. erectosquamosus* have been previously reported to be from Thailand by Wisitrassameewong et al. (2012) and Zhao et al. (2016), respectively. *Agaricus pallidobrunneus* has been reported from China (Zhao et al., 2016), however, it has now been found for the first time in Thailand. Thus, to our knowledge, the *Agaricus* species recorded in Thailand has been raised to 62 species, 13 sections in five subgenera by the morphological and molecular evidence. Nevertheless, 13 *Agaricus* species listed by Chandrasrikul et al. (2011) require further confirmation by molecular data.

## Data Availability Statement

The datasets presented in this study can be found in online repositories. The names of the repository/repositories and accession number(s) can be found in the article/Supplementary Material.

## Author Contributions

CJ, JK, and NS: conceptualization and resources. CJ, JK, SV, and NS: methodology, formal analysis, and writing-review and editing. SV and CJ: software. NS and SL: validation. CJ, JK, and SV: investigation, data curation, and writing-original draft. NS and SL: supervision. All authors read, revised, and approved the final manuscript.

## Conflict of Interest

The authors declare that the research was conducted in the absence of any commercial or financial relationships that could be construed as a potential conflict of interest.

## Footnotes

1 http://blast.ncbi.nlm.nih.gov

## Supplementary Material

Supplementary Table 1 Names, voucher numbers, countries and the corresponding GenBank accession numbers of the taxa used in the phylogenetic analyses. The sequences obtained in this study are in bold and superscript “T” refers to a type.
