# Supplementary material for: Multigene Phylogeny and Morphology Reveal Three Novel Species and a Novel Record of Agaricus From Northern Thailand
Source: Front Microbiol. 2021 Jun 21;12:650513. doi: 10.3389/fmicb.2021.650513 (PMC8256166; doi:10.3389/fmicb.2021.650513)
Supplement: Supplementary Table 1 — Names, voucher numbers, countries and the corresponding GenBank accession numbers of the taxa used in the phylogenetic analyses. The sequences obtained in this study are in bold and superscript “T” refers to a type. [file Table_1.pdf]

TABLE 1. Names, voucher numbers, countries and the corresponding GenBank accession numbers of the taxa used in the phylogenetic analyses. The sequences obtained in this study are in bold and superscript "T" refers to type.

***Agaricus* species**	**Specimen/voucher**	**Country**	**GenBank accession number**			**Reference**
			**ITS**	**LSU**	***tef*1**	
A. abruptibulbus	LAPAG524	Czech Republic	KJ548132	–	–	Gui et al. (2015)
A. abruptibulbus	ZRL2012005	China	KT951356	KT951460	KT951626	Zhao et al. (2016)
A. aridicola	LAPAG589	Spain	KT951331	KX084027	KX198081	Zhao et al. (2016) and Chen et al. (2017)
A. armandomyces	ZRL2015992^{T}	China	KX684860	KX684882	KX684906	He et al. (2017)
A. arrillagarum	LAPAG810	Spain	KF447900	KX083985	KT951592	Zhao et al. (2016) and Chen et al. (2017)
A. arvensis	MA Fungi 80999	Spain	KF114474	–	–	Gui et al. (2015)
A. badioniveus	LD2012131	Thailand	KU975117	–	KX198072	Chen et al. (2017)
A. bingensis	C3155	Togo	KJ540950	–	–	Chen et al. (2015)
A. bohusii	LAPAG562	Spain	KM657928	KR006613	KR006641	Zhou et al. (2016)
A. brunneolutosus	MS514^{T}	China	KU975111	KX084006	–	Chen et al. (2017)
A. brunneopunctatus	ADK2564	Benin	JF514518	–	–	Chen et al. (2015)
A. brunneosquamulosus	ZRL133	Thailand	KT951344	KT951505	KT951656	Zhao et al. (2016)
A. caballeroi	AH44503	Spain	KJ575605	–	–	Parra et al. (2014)
A. callacii	AH42929^{T}	Spain	KF447899	KX083984	KX198051	Chen et al. (2017)
A. campbellensis	GAL9420^{T}	New Zealand	DQ232644	DQ232657	–	Geml et al. (2007)
A. campestris	LAPAG370^{T}	Spain	KM657927	KR006607	KR006636	Zhao et al. (2016)
A. catenatus	ZRL2012104^{T}	China	KX657023	KX656963	KX684957	He et al. (2017)
A. cerinipileus	ZRL2012001^{T}	China	KX657021	KX656957	KX684953	He et al. (2017)
A. cf. *inoxydabilis*	LAPAF1	Togo	JF727841	–	–	Zhao et al. (2011)
A. cf. *tenuivolvatus*	LAPAG714	Spain	KJ548134	–	–	Gui et al. (2015)
A. chartaceus	H6271	Australia	JF495048	–	–	Lebel and Syme (2012)
A. chiangmaiensis	NTS113	Thailand	JF514531	–	–	Zhao et al. (2011)
A. coccyginus	ZRL2014354	China	–	KX656936	KX684998	He et al. (2017)

A. collegarum	L0608780^{T}	France	KP241113	–	–	Parra et al. (2015)
A. collegarum	L0608780	France	KP241114	–	–	Parra et al. (2015)
A. colpetei	TL2424^{T}	Australia	JX984565	–	–	Lebel (2013)
A. columellatus	MIN938394	USA	KJ912899	–	–	Bates et al. (2016)
A. comtulus	LAPAG303	Spain	KU975078	KX083986	KX198052	Chen et al. (2017)
A. crassisquamosus	ZRL2012607^{T}	China	KT951376	KT951510	KT951645	Zhao et al. (2016)
A. crocodilinus	LAPAG223	Spain	KJ548127	–	–	Gui et al. (2015)
A. desjardinii	WZR2012907	China	KM657901	KT951474	KT951644	Dui and Zhao (2015) and Zhou et al. (2016)
A. desjardinii	WZR20128212^{T}	China	KM657900	–	–	Dui and Zhao (2015)
A. dilatostipes	ZRL2014450	China	KX656999	KX656941	KX685003	He et al. (2017)
A. dolichopus	ZRL2012715	China	KT951382	KT951502	KT951573	Zhou et al. (2016)
A. dolichopus	ZRL2014120	China	KT951433	–	–	Zhou et al. (2016)
A. dulcidulus	PRM909627	Czech Republic	KF447894	–	KX198064	Chen et al. (2017)
A. duplocingulatoides	CUH AM602	India	MH511804	–	–	Tarafder et al. (2018)
A. duplocingulatus	ZRL2012267	China	KT951368	KT951504	KT951655	Zhao et al. (2016)
A. eburneocanus	MURU5927^{T}	Australia	JF495063	NG042583	–	Lebel and Syme (2012)
A. edmondoi	LAPAG412	Spain	KT951326	KT951481	KT951590	Zhao et al. (2016)
A. elongatestipes	ZRL2013271^{T}	China	KX657002	KX656946	KX684975	He et al. (2017)
A. erectosquamosus	**SDBR-CJ0131**	**Thailand**	**MW255807**	**MW255831**	**MW264839**	**This study**
A. erectosquamosus	**SDBR-CJ0032**	**Thailand**	**MW255804**	**MW255803**	**MW264838**	**This study**
A. erectosquamosus	**SDBR-NK0080**	**Thailand**	**MW255805**	**MW255806**	**MW264840**	**This study**
A. erectosquamosus	LD2012165^{T}	Thailand	KT951338	KT951509	KT951565	Zhao et al. (2016)
A. erythrosarx	MURU6080	Australia.	JF495068	–	–	Lebel and Syme (2012)
A. essettei	ZRL2012599	China	KT951374	KT951514	KT951625	Zhao et al. (2016)
A. excellens	RWK1929	USA	AY484682	–	–	Geml et al. (2004)
A. fiardianus	F2389	France	JF727851	–	–	Zhou et al. (2016)
A. fimbrimarginatus	LD201250^{T}	Thailand	KU975119	KX084017	KX198076	Chen et al. (2017)
A. fissuratus	LAPAG 488	Spain	JQ824135	–	–	Foulongne et al. (2012)
A. flammicolor	LD201502^{T}	Thailand	KU975114	KX084009	KX198042	Chen et al. (2017)
A. flavopileatus	MS596^{T}	China	KU975121	KX084022	KX198078	Chen et al. (2017)

A. flocculosipes	ZRL2012105	China	KT951365	KT951463	KT951618	Zhao et al. (2012b)
A. friesianus	LAPAG592	France	KT951316	KX083992	KT951594	Zhao et al. (2016) and Chen et al. (2017)
A. fulvoaurantiacus	LD201404^{T}	China	KU975107	KX084002	KX198069	Chen et al. (2017)
A. gemlii	AH44510^{T}	Spain	KF447891	KX083989	–	Chen et al. (2017)
A. gemloides	ZRL2012017	China	KT633274	KX656959	KX684955	He and Zhao (2015) and He et al. (2017)
A. gemloides	ZRL2014084^{T}	China	KT633271	KX641405	KX684986	He and Zhao (2015) and He et al. (2017)
A. globosporus	ZRL2012652	China	KX657036	KX656976	KX684967	He et al. (2017)
A. heinemannianus	LAPAG302	Spain	KF447906	–	KX198056	Chen et al. (2017)
A. indistinctus	LAPAG478	Spain	KF114475	–	–	Gui et al. (2015)
A. jacobi	AH44505^{T}	Spain	NR158300	KX083996	KX198061	Chen et al. (2017)
A. jingningensis	ZRL20151562^{T}	China	KX684877	KX684895	KX684917	He et al. (2017)
A. kerriganii	LAPAG808	Spain	KT951306	KT951442	KT951589	Zhao et al. (2016)
A. kunmingensis	ZRL2012015	China	KT951361	KT951506	KT951642	Zhou et al. (2016)
A. kunmingensis	ZRL2012007	China	KT951427	–	–	Zhou et al. (2016)
A. lannaensis	**SDBR-CJ0192**	**Thailand**	**MW255680**	**MW255681**	**MW264836**	**This study**
A. lannaensis	**SDBR-NK0564^{T}**	**Thailand**	**MW255657**	**MW255674**	**MW264834**	**This study**
A. lannaensis	**SDBR-NK0584**	**Thailand**	**MW255738**	**MW262926**	**MW264835**	**This study**
A. laparrae	SFSU F 020928 ^{T}	California	NR144983	–	–	Unplublished
A. laparrae	RWK 2039	California	KJ577975	–	–	Unplublished
A. longistipes	HKAS81066^{T}	China	NR151748	–	–	Gui et al. (2015)
A. luteofibrillosus	ZRL2012359	China	KU245978	KX656967	KX684959	He et al. (2017)
A. luteomaculatus	CA331	France	KF447901	–	KX198053	Chen et al. (2017)
A. luteopallidus	LD2012113	Thailand	KU975124	KX084026	KX198080	Chen et al. (2017)
A. macrocarpus	LAPAG575	Spain	KJ548129	–	–	Gui et al. (2015)
A. mangaoensis	ZRL2010056^{T}	China	KX657042	KX656956	KX684946	He et al. (2017)
A. marisae	LAPAG138^{T}	Spain	KU975083	KX083998	KX198065	Chen et al. (2017)
A. masoalensis	ZTMyc57137^{T}	Switzerland	KP282686	–	–	Parra et al. (2015)
A. masoalensis	ZTMyc57138	Switzerland	KP282687	–	–	Parra et al. (2015)

A. matrum	LAPAG817[T]	Spain	KF447896	KX083991	KX198058	Chen et al. (2017)
A. megacystidiatus	MFLU 2012 1004	Thailand	KF305946	–	–	Karunarathna et al. (2014)
A. megalocarpus	HKAS71717[T]	China	NR151747	–	–	Gui et al. (2015)
A. megalosporus	MFLU100774[T]	Thailand	NR119951	KX084019	KX198077	Schoch et al. (2014) and Chen et al. (2017)
A. microviolaceus	ZRL2012718[T]	China	KX657033	KX656980	KX684971	He et al. (2017)
A. minipurpureus	ZRL2010058[T]	China	KX657043	KX656953	KX684947	He et al. (2017)
A. neimengguensis	ZRL20151845[T]	China	KX684870	KX684902	KX684924	He et al. (2017)
A. niveogranulatus	MFLU 2011 1329	Thailand	KJ540960	–	–	Chen et al. (2015)
A. padanus	WZR20128213	China	KM657902	–	–	Dui and Zhao (2015)
A. padanus	WZR2012903	China	KM657903	–	–	Dui and Zhao (2015)
A. pakistanicus	LAH35299	Pakistan	MG669256	–	–	Bashir et al. (2018)
A. pallens	LAPAG926	Sweden	KT951315	–	KT951591	Chen et al. (2017)
A. pallidobrunneus	**SDBR-NK0368**	**Thailand**	**MW255649**	**MW255654**	**MW264829**	**This study**
A. pallidobrunneus	ZRL2012358[T]	China	KT951370	KT951471	KT951566	Zhao et al. (2016)
A. parvibicolor	LD2012116[T]	Thailand	KP715162	–	–	Liu et al. (2015)
A. parvibrunneus	ZRL20161053[T]	China	MG137001	MG196345	MG196351	He et al. (2018b)
A. patris	LD201224[T]	Thailand	KU975118	KX084012	KX198073	Chen et al. (2017)
A. pseudoerectosquamosus	**SDBR-CJ0108**	**Thailand**	**MW255655**	**MW255656**	**MW264831**	**This study**
A. pseudoerectosquamosus	**SDBR-NK0064[T]**	**Thailand**	**MW255736**	**MW255737**	**MW264830**	**This study**
A. pseudolutosus	LAPAG454	Spain	KT951329	KT951453	KT951602	Zhao et al. (2016)
A. pseudominipurpureus	ZRL2013341[T]	China	MG137000	MG196343	MG196350	He et al. (2018b)
A. pseudopurpurellus	ZRL2014063[T]	China	KX656988	KX641404	KX684985	He et al. (2017)
A. purpurellus	LAPAG944	Czech Republic	KU975076	KX083994	KX198060	Chen et al. (2017)
A. purpureofibrillosus	ZRL3080[T]	Thailand	JF691542	KX084021	–	Zhao et al. (2011) and Chen et al. (2017)
A. purpurlesquameus	MFLU17 1306[T]	Thailand	NR157484	–	–	Hyde et al. (2017)
A. robustulus	CA847[T]	Thailand	KU975086	KX084034	KX198039	Chen et al. (2017)
A. rufifibrillosus	ZRL20151536[T]	China	KX684878	KX684893	KX684915	He et al. (2017)
A. rufipileus	ZRL2014140[T]	China	KX656991	KX656937	KX684991	He et al. (2017)
A. sodalis	LD2012159[T]	Thailand	KP715161	KX084014	KX198074	Liu et al. (2015)
A. sordidocapus	MFLU 2012 0881	Thailand	KJ540946	–	–	Chen et al. (2015)

A. sp.	MS386	China	KU975113	KX084008	KX198044	Chen et al. (2017)
A. sp.	ZRL2010079	China	KX657046	KX656951	KX684950	He et al. (2017)
A. sp.	CA935	Thailand	KU975085	KX084036	KX198034	Chen et al. (2017)
A. sp.	ZRL2010099	China	KM657882	KT951479	KT951564	Zhao et al. (2016)
A. sp.	LD2012162	Thailand	KT951337	KT951493	KT951563	Zhao et al. (2016)
A. sp.	CA820	Thailand	JF727861	–	–	He et al. (2018)
A. sp. 3	NTT117	Thailand	JF514534	–	–	Chen et al. (2015)
A. sparsisquamosus	LAH35201	Pakistan	KY741892	KY741897	–	Bashir et al. (2018)
A. stevensii	FS 06 02 09	USA	KJ877785	–	–	Kerrigan (2016)
A. subrufescens	GY 128883	China	KJ755634	–	–	Gui et al. (2015)
A. subrufescens	**SDBR-NK0079**	**Thailand**	**MW255802**	**MW255801**	**MW264837**	**This study**
A. subrufescens	ZRL2012722	China	KT951383	KT951451	KT951632	Zhao et al. (2016)
A. subsaharianus	ADK4732	Burkina–Faso	JF440300	–	–	Ibrahim et al. (2010)
A. sylvaticus	ZRL2012013	Thailand	KT951360	KT951500	KT951570	Zhao et al. (2016)
A. sylvaticus	ZRL2012568	China	KT951371	KT951501	KT951568	Zhao et al. (2016)
A. thailandensis	**SDBR-CJ0118^{T}**	**Thailand**	**MW255675**	**MW255677**	**MW264832**	**This study**
A. thailandensis	**SDBR-CJ0225**	**Thailand**	**MW255678**	**MW255679**	**MW264833**	**This study**
A. toluenolens	MFLU 2014 0026	Thailand	KJ540948	–	–	Chen et al. (2015)
A. variabiliclor	ZRL4007	Thailand	KT951439	–	–	Zhou et al. (2016)
A. variabiliclor	ZRL4012	Thailand	KT951440	–	–	Zhou et al. (2016)
A. variabiliclor	ZRL4002	Thailand	KT951438	–	–	Zhou et al. (2016)
A. wariatodes	TWM1589	Australia	JF495052	JF495030	–	Lebel and Syme (2012)
A. yanzhiensis	ZRL20162082^{T}	China	MG137003	MG196346	–	He et al. (2018b)
